# VariFAST: a variant filter by automated scoring based on tagged-signatures

**DOI:** 10.1186/s12859-019-3226-2

**Published:** 2019-12-30

**Authors:** Hang Zhang, Ke Wang, Juan Zhou, Jianhua Chen, Yizhou Xu, Dong Wang, Xiaoqi Li, Renliang Sun, Mancang Zhang, Zhuo Wang, Yongyong Shi

**Affiliations:** 1 Bio-X Institutes, Key laboratory for the Genetics of Developmental and Neuropsychiatric Disorders (Ministry of Education), Shanghai Jiao Tong University, Shanghai, 200030 China; 2 Shanghai Key Laboratory of Psychotic Disorders, Shanghai Mental Health Center, Shanghai Jiao Tong University School of Medicine, Shanghai, 200030 People’s Republic of China; 3 DYnastyGene Biotech Co. Ltd., Building 25, No.10688 Bei Qing Road, Qingpu District, Shanghai, 201700 People’s Republic of China

**Keywords:** Variant filtering, Automated scoring, Tagged-signatures, Germline mutation, Somatic mutation

## Abstract

**Background:**

Variant calling and refinement from whole genome/exome sequencing data is a fundamental task for genomics studies. Due to the limited accuracy of NGS sequencing and variant callers, IGV-based manual review is required for further false positive variant filtering, which costs massive labor and time, and results in high inter- and intra-lab variability.

**Results:**

To overcome the limitation of manual review, we developed a novel approach for **Vari**ant **F**ilter by **A**utomated **S**coring based on **T**agged-signature (VariFAST), and also provided a pipeline integrating GATK Best Practices with VariFAST, which can be easily used for high quality variants detection from raw data. Using the bam and vcf files, VariFAST calculates a *v-score* by sum of weighted metrics causing false positive variations, and marks tags in the manner of keeping high consistency with manual review, for each variant. We validated the performance of VariFAST for germline variant filtering using the benchmark sequencing data from GIAB, and also for somatic variant filtering using sequencing data of both malignant carcinoma and benign adenomas as well. VariFAST also includes a predictive model trained by XGBOOST algorithm for germline variants refinement, which reveals better MCC and AUC than the state-of-the-art VQSR, especially outcompete in INDEL variant filtering.

**Conclusion:**

VariFAST can assist researchers efficiently and conveniently to filter the false positive variants, including both germline and somatic ones, in NGS data analysis. The VariFAST source code and the pipeline integrating with GATK Best Practices are available at https://github.com/bioxsjtu/VariFAST.

## Background

With the rapid development of sequencing technologies and the decrease of the costs, a tremendous amount of genome-wide data, including whole exome and genome sequencing data, expression profile data, single nucleotide polymorphism (SNP) and copy number spectrum, as well as functional experimental data, are available for biomedical researches. Different sequencing workflows and platforms have distinctive advantages, e.g. there are many popular variations calling tools, such as Mutect2 [1], SomaticSniper [2], Strelka [3], VarScan2 [4] and HaplotyperCaller [5]. However, false positive alterations still often survive in the final results. In order to acquire high-quality mutations from raw data, the automated pipelines were used to identify and wipe out many false positive calls resulting from sequencing errors, misalignment of reads, and other factors [6, 7]. However, to avoid being misled by inaccurate detection of variants, additional manual refinement of alterations is crucial for minimizing false positives and determining candidate variations for further disease studies.

As for germline mutations, it typically involves employing Variant Quality Score Recalibration (VQSR) to produce callsets ready for downstream genetic analysis via using resources of known variation, truthsets and other metadata to assess and improve the accuracy of the results. However, VQSR requires a large scale of samples and tends to work well enough with at least one whole genome or 30 exomes. Anything smaller than that scale is likely to run into difficulties. As for somatic mutations, FilterMutectCalls is recommended to filter based on sequence context artifacts. However, additional filtering, for instance, manually reviewing is also necessary for further studies, which extends from deciphering call record annotations to the nitty-gritty of reviewing read alignments using a visualizer.

The Integrative Genomics Viewer (IGV), a lightweight visualization tool that enables intuitive real-time exploration of variants, were developed to handle various types of data [8, 9]. Moreover, minimizing false positive alterations via IGV also becomes a traditional method to manually examine sequencing data. Nonetheless, it is obviously not an effective method and has a personal preference. It is necessary to develop more efficient methods and tools for such purposes. In a recent publication, an SOP has been put forwarded, which includes the summarized 19 factors accounting for false positive variants, and provides guideline for variants refinement [10]. In addition, there are benchmark sequencing data from GIAB (the Genome in a Bottle Consortium) for the CEPH/HapMap genome HG001 (NA12878) have been widely used to develop, optimize, and demonstrate the performances of sequencing and bioinformatics methods [11]. Therefore, high-confidence calls of GIAB datasets also can be used for evaluating the reliability and accuracy of variant refinement methods.

In this study, we developed a novel approach for automated filtering false positive variations, called VariFAST, which is based on both weighted score and machine learning model. By using the bam and vcf files, VariFAST calculates a *v-score* by sum of weighted metrics causing false positive variations, and marks tags according to the SOP [10], in the manner of keeping high consistency with existing knowledge, for each variant. We validated the performance of VariFAST for germline variant filtering using the benchmark sequencing data from GIAB, and also for somatic variant filtering using sequencing data of penile squamous cell carcinoma and pituitary adenomas by our Lab. This pipeline of variant refinement is substantially useful for researchers to get a more comprehensive understanding of the pathogenic mechanism of diseases.

## Materials and methods

### Overall strategy of VariFAST approach for automated variant refinement

**Fig. 1:**
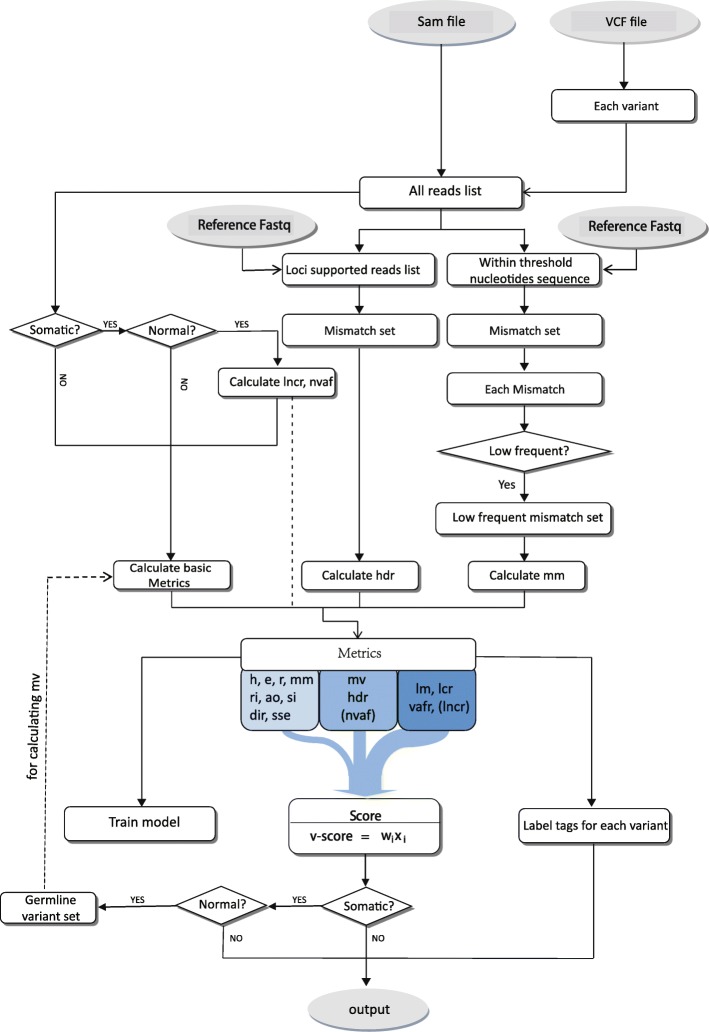
The overall design of VariFAST

Figure 1 illustrates the strategy and procedure of VariFAST approach. VariFAST contains four major modules: metric calculating, tag-marking, *v-score* evaluating, and model training.

The Basic metrics contains *h, e, r, ri, ao, si, dir, sse, mv, lm, lcr, vafr.* The metrics in brackets are used for somatic variants filtering. The input and output files were highlighted in grey.

The first step is metric calculating, in which all reads are stored in the list according to their original position on chromosomes. All mismatches around the variant locus are considered for further metric calculating. For germline variants, 16 metrics are calculated based on reads list. For somatic variants, it will be divided into two partition. For normal track, potential germline variants on the locus are found by *v-score* and two additional metrics are computed. Then, reads list together with potential germline variants are used to calculate metrics on cancer track. Finally, all 16 metrics for germline variants and 18 metrics for somatic variants are used for tag-marking, *v-score* evaluating and model training, each step will be explained in more details (see 2.4, 2.5, 2.6).

We implemented the VariFAST approach with python and developed an easy-to-use tool for users to filter variants. We also provide a pipeline integrating GATK Best Practices Pipeline with VariFAST, which can be convenient for users to get high quality variants from raw sequencing data more efficiently.

The detailed flowchart is shown in Additional file 1: Figure S1, and this pipeline can also be downloaded from Github.

### Definition and calculation of metrics for variant filtering

We proposed 16 quantitative metrics for germline variant filtering and 18 for somatic variant filtering generally based on SOP of IGV manual review [10], as follows:

1. Low coverage Risk
  1 $$ lcr=1-\frac{\min \left( thr,d\right)}{thr} $$

Where *d* is the total read counts covering the specific locus. According to different sequencing data, *thr* represents the users’ requirement for minimal read counts. *Lcr* ranges from 0 to 1, while 0 means the variant passes the quality control of low coverage.

2. Variant allele frequency risk
  2 $$ vafr=1-\frac{d_m}{d} $$

Where *d*_*m*_ is the depth of the variant.

3. Near Head Rate
  3 $$ nh=\frac{d_h}{d_m} $$
  *d*_*h*_ is the number of supported reads whose distance between read’s head and variant locus is under threshold (default 5).
4. Near End Rate
  4 $$ ne=\frac{d_e}{d_m} $$
  *d*_*e*_ is the number of supported reads whose distance between read’s end and variant locus is under threshold (default 5).
5. Near Insertion Rate
  5 $$ ni=\frac{d_{insert}}{d_m} $$
  *d*_*insert*_ is the number of events where the neighbor of the variant is an insertion.
6. Near deletion Rate
  6 $$ nd=\frac{d_{del}}{d_m} $$

*d*_*del*_ is the number of events where the neighbor of the variant is a deletion.

7. Same Start/End Read Rate
  7 $$ sse=\frac{\mathit{\max}{d}_s}{d_m} $$
  *d*_*s*_ is the number of supported reads who have the same start and end.
8. Directional Read Rate
  8 $$ dir=\frac{\mathit{\max}{d}_d}{d_m} $$
  *d*_*d*_ is the number of supported reads who have the same direction.
9. Low Mapping Read Rate:
  9 $$ lm=\frac{d_l}{d_m} $$
  *d*_*l*_ is the number of variants supported reads whose mapping quality is under threshold (default 10).
10. Multiple Mismatched Rate
  10 $$ mm=\frac{\left(\sum i\in T{v}_i\right)\times 100}{n} $$
  Where *T* is the set of mismatches whose frequency are under threshold (default 0.05), *v*_*i*_ represents the depth of mismatch *i* and *n* means the number of nucleotides within 10 bp upstream and 10 bp downstream.
11. Multiple Variants Rate
  11 $$ mv=\frac{d_{mv}}{d_m} $$
  Where *d*_*mv*_ is the number of alleles at the same position.
  Notably, for somatic variants, the program will identify potential germline variants first via using normal track according to *v-score* (see 2.3) and then count the *d*_*mv*_ according to the tumor track, excluding germline variant with same position detected before.
12. High Discrepancy Region
  12 $$ hdr=\left\{\begin{array}{c}\sum \limits_i{HR}_i,{HR}_{max}\ge threshold\\ {}{HR}_{max},{HR}_{max}< threshold\end{array}\right. $$
  *HR* $\left( HR=\frac{2h}{d_m+{d}_i}\right)$ is calculated for all mismatches located on same reads with candidate variant, where *d*_*i*_ the depth of mismatch and *h* is the counts of mismatch and candidate variant occurring on same read. *HR*_*max*_ is the maximum of all *HR*. Particularly, the value of *hdr* might be greater than 1 as more than one mismatch appeared high concordant with candidate variant (default *HR* ≥ 0.9).
13. Short Inserts Rate
  13 $$ si=\frac{d_{si}}{d_p} $$
  Tag Short Inserts (SI) is frequently in data derived from archival material (FFPE) or other source material with small DNA fragment [12], where almost all variants appeared in the overlapping region of the two read fragments. Metric *si* is set to catch this tag, where *d*_*si*_ is the number of variants appeared in the overlapping region of both read fragments and *d*_*p*_ is the counts of the paired supported reads.
14. Repeat *r*: when candidate variant is near repeat region, *r* will be 1.
15. Repeat Insert *ri*: when insertion is near repeat region, and insert nucleotides are the same as the minimum repeat unit, *ri* will be 1.
16. Large Insert *li*: when the length of insert fragment reach threshold (default 20) and nucleotides of the fragments are the same as the near nucleotides of reference, *li* will be 1.

As for somatic variant, there are two additional metrics.

17. Normal Variant allele frequency for somatic variation
  14 $$ nvaf=\frac{d_n}{d_{normal}} $$
  Where *d*_*n*_ is the depth of variants in normal track, and *d*_*normal*_ is total read counts covering the locus in normal track.
18. Low normal coverage risk for somatic variation
  15 $$ lncr=1-\frac{\mathit{\min}\left({thr}_n,{d}_{normal}\right)}{thr_n} $$
  Where *thr*_*n*_ represents the users’ requirement for minimal read counts in normal track.

All metrics mentioned above ranged from 0 to 1 except *mv, hdr* and *mm*.

### Quantification of the variant score by sum of weighted metrics

We proposed an indicatrix named variant score (*v-score*) to comprehensively consider the effects of all metrics. The specific formula for calculating *v-score* is as follow, where *w* means the weight of each metric, and *x* is the value of metric calculated above.

16 $$ v=\sum {w}_i{x}_i $$

On the basis of prior knowledge [10], the 18 metrics are partitioned into three importance levels with different weights. The most important metrics including *lcr, vafr, lm, ni* and *nd*, are regarded as level3, and the weights are assigned as 3. The level2 including *lncr*, *mv* and *hdr* are also crucial for false variant filtering, whose weight values are 2. The remaining metrics are regarded as level1, which may not be dominant metrics for refinement. In addition, each metric’s weight is not fixed and can be changed to fulfill different kinds of applications.

### Assignment of tags according to metrics

*Barnell* et al. summarized 19 factors worth being concerned when visualizing the variant through IGV: Adjacent Indel (AI), Ambiguous Other (AO), Directional (DIR), Dinucleotide Repeat (DN), Mononucleotide (MN), Ends (EN), Tandem Repeat (TR), High Discrepancy Region (HDR), Low Count Normal (LCN), Low Count Tumor (LCT), Low Mapping (LM), Low Variant Frequency (LVF), Multiple Mismatches (MM), Multiple Variants (MV), No Coverage in Normal (NCN), Short Inserts Only (SIO), Short Inserts (SI), Same Start/End (SSE), Tumor in Normal (TN) [10]. Whereas in this study, we combined the DN, TR, MN together as a new tag Repeat Region (RR), SIO and SI are combined as tag SI. AI is divided into two new tags Neat Insertion (NI) and Near Deletion (ND). Two new tags HE (Head) and RI (Repeat Insert) were added, where HE represents nearly all variants are near to head of reads and RI means insertion happening on repeat region and inserted nucleotides are same as the minimum repeat units. To summarize, we proposed a more reasonable and quantitative standard including 19 tags for variant filtering. These tags are automatically marked according to the corresponding metrics defined above, and the standard is shown in Table 1.

**Table 1:** Description of tags used to annotate variants and the standards of corresponding metrics

Tag	Standard of metric	Description
*LC*	*lcr* > 0	Low coverage in track
*LVF*	$vafr\ge 1-{thr}_{vaf}^{\ast }$	Low variant allele frequency
*LM*	(*lm* ≥ 0.2) ∧ (*d*_*l*_ ≥ 2)	Low mapping quality
*MM*	*mm* ≥ 1	Too many mismatches around variant
*HDR*	*hdr* ≥ 1	The variant is supported by reads that have other recurrent mismatches
*HE*	*nh* ≥ 0.9	Near head
*EN*	*ne* ≥ 0.9	Near end
*NI*	*ni* ≥ 0.9	Near insertion
*ND*	*nd* ≥ 0.9	Near deletion
*D*	*dir* ≥ 0.9	Almost supported reads with the same direction
*SSE*	*sse* ≥ 0.9	Almost supported reads with the same start and end
*MV*	*(mv* ≥ 0.2) ∧ (*d*_*mv*_ ≥ 2)	Variant locus has read support for different alleles
*RR*	*r* = 1	Near repeat region
*RI*	*ri* = 1	Insertion and near repeat region and insert nucleotides are the same as minimum repeat units of reference
*AO*	*li* = 1	Big insertion and insert nucleotide are the same as the reference
*SI*	*si* ≥ 0.9	Supported reads are short insert
*(NCN)*	*lncr* = 1	No coverage in normal track
*(LCN)*	1 > *lncr* > 0	Low coverage in normal track
*(VN)*	(*nvaf* ≥ 0.1 × *thr*) ∧ (*d*_*normal*_ ≥ 2)	Variant occur in normal track

*thr*_*vaf*_ is the requirement for minimal variant allele frequency set by users. The tags in brackets are used for somatic variants

### Evaluation of variant refinement by v-score

The *F*_*β*_ score was calculated as ${F}_{\beta }=\frac{\left(1+{\beta}^2\right)\times P\times R}{\left({\beta}^2\times P\right)+R}$ to select the most appropriate *v-score*. The value of *β* influence the balance between precision (P) and recall (R), *β*> 1 demonstrates that recalls plays more important role, while if *β*< 1, the precision becomes a major effect.

### Machine learning model for advanced variant filtering

Supervised machine learning method usually behaves well in a situation with big data, which requires little prior knowledge. XGBoost is an effective and widely used machine learning method, which is based on Gradient Boosting framework [13]. Here, we used XGBOOST to construct a model for false positive variant filtering.

All metrics calculated above were used to train the model with binary-logistic as objective function. The default values were selected for almost all hyperparameters other than learning rate (eta), minimum loss reduction for a further partition (gamma) and the maximum depth of the tree (max_depth). The large gamma makes the model conservative and results in underfitting, oppositely, increasing max_depth may more likely lead to overfitting. Grid search with ten-fold cross-validation was performed for hyperparameters selection while eta was chosen from (0.01; 0.05; 0.1), gamma from (0.1; 1; 10), max_depth from (3; 6; 9).

### Sequencing data with high-confidence germline variant annotation

Six datasets sequenced for four samples (HG001, HG002, HG003, HG004) which contain high-confidence benchmark compiled by GIAB were used in this study [11, 14, 15]. The benchmark set was generated by integrating multi-datasets from 5 different sequencing platforms and could be retrieved from (ftp://ftp-trace.ncbi.nlm.nih.gov/giab/ftp/release/). Our lab also sequenced the HG001 sample using Illumina Xten platform, and generated two exome sequencing datasets HG001_b and HG001_c. Samples HG002, HG003 and HG004 are Ashkenazim Trio from GIAB. The specific information of datasets is shown in Table 2. All sequencing data are mapped using BWA MEM [17, 18] and called variants by GATK HaplotypeCaller.

**Table 2:** Description of sequencing datasets for germline variant refinement

ID	Sample	Platform	Coverage	Source
HG001_a	HG001	Illumina Hiseq 2000	88	1000 genomes
HG001_b	HG001	Illumina Xten	87	Our lab
HG001_c	HG001	Illumina Xten	85	Our lab
HG002	HG002	Illumina Hiseq 2500	226	GIAB
HG003	HG003	Illumina Hiseq 2500	193	GIAB
HG004	HG004	Illumina Hiseq 2500	214	GIAB

data set of HG001_a is available on the ftp site of 1000 genomes [16] (ftp://ftp.1000genomes.ebi.ac.uk/vol1/ftp/). data sets of HG001_b and HG001_c are sequenced by our lab. Data sets of Ashkenazim Trio including HG002, HG003 and HG004 are available on the ftp site of GIAB (ftp://ftp-trace.ncbi.nlm.nih.gov/giab/ftp/data/)

### Sequencing data for somatic variant refinement

Sequencing data of two different cancers conducted by our research team were used to show the application of VariFAST for somatic variant refinement. The first one is three paired whole exome datasets sequencing for penile squamous cell carcinoma cases, which were collected in the Affiliated Hospital of Qingdao University. The other one is whole exome datasets for 136 Pituitary adenomas (PAs) published in our previous study [19].

## Results

### Variant refinement performance on germline variants

#### Summary of variants called from different standard sequencing samples

In order to assess whether VariFAST method is able to improve the accuracy of variant refinement, we aligned the sequence data of samples HG001, HG002, HG003, and HG004 by different Illumina sequencers to the reference genome (hg19) using BWA MEM, and used GATK HaplotypeCaller to call the candidate variants. The candidate variants consistent with high confidence variants annotated in GIAB are positive, while others are regarded as negative (Table 3).

**Table 3:** The number of variants with true positive and true negative confidence in different standard sequencing samples

Sample ID	Positive variants	Negative variants	Total variants
HG001_a	28,680	5269	33,949
HG001_b	46,068	10,538	56,606
HG001_c	46,031	10,453	56,484
HG002	36,383	7769	44,152
HG003	35,879	8175	44,054
HG004	36,277	8051	44,328
All	229,318	50,255	279,573

#### Performance of variant filtering by tags

**Fig. 2:**
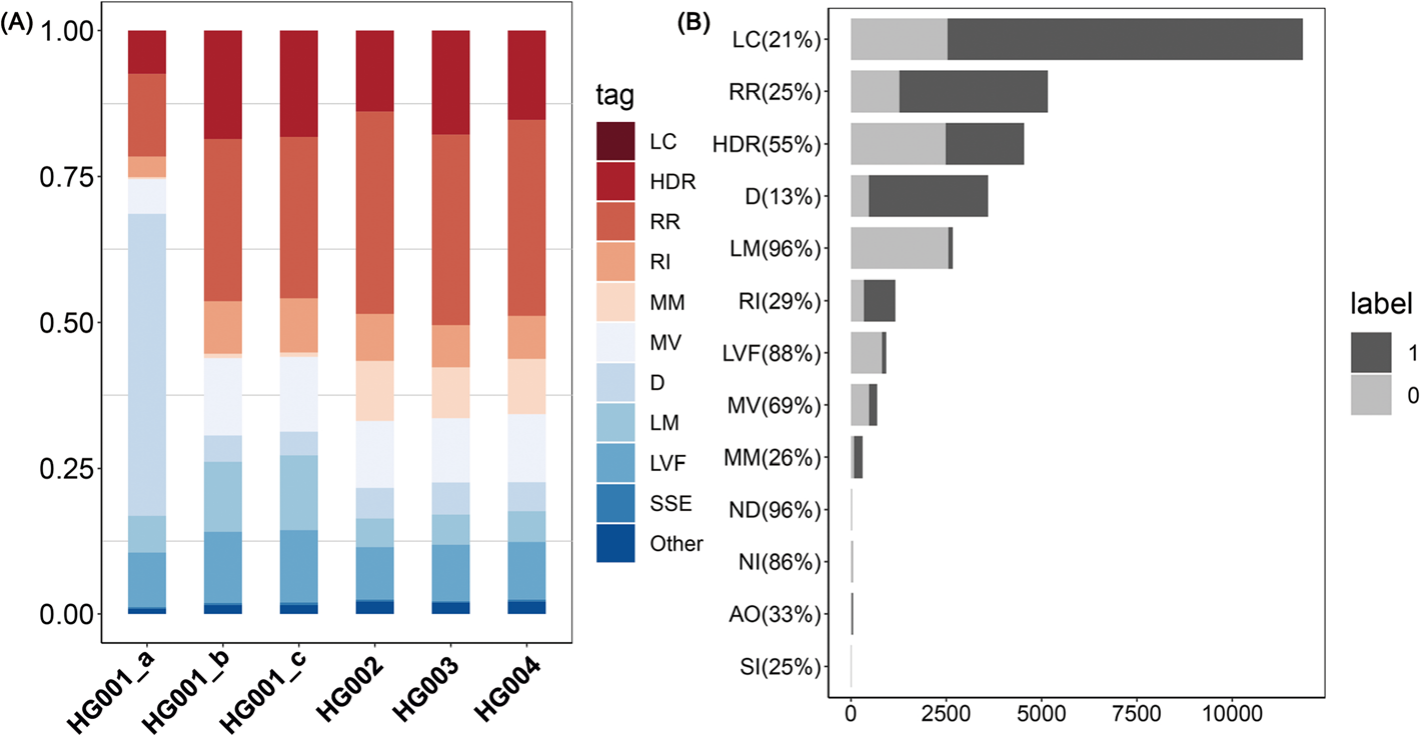
The influence of different tags on differentiation of negative variants. **a** The proportion of tags in six datasets of standard sequencing samples. The Y coordinate represents the tag proportion, which is counted as the number of variants having each specific tag divided by the total number of variants having any of 19 tags. (Note that one variant may have several different tags). **b** The effectiveness of tags for determining negative variants. 1 represents the variant is true positive, and 0 represents the variant is negative without high confidence in the benchmark. The percentage is the proportion of negative variants having the corresponding tags

For all of the 279,573 germline variants, we used VariFAST to calculate 16 metrics, and then get the *v-score* and mark the tags. The distributions of tags showed slightly different patterns among the six standard sequencing datasets (Fig. 2a). While HG001 from 1000 genome has a higher ratio in tags LC and D than other datasets, the samples of HG002, HG003, and HG004 have a higher ratio in tag MM. The variants marked by only one tag (RI only with RR are also considered because RR is the prerequisite of RI) are selected to validate the effectiveness of tags, including 2671 variants with LM; 3596 variants with D; 687 variants with MV; 305 variants with MM; 925 variants with LVF; 5164 variants with RR; 4542 variants with HDR; 11,852 variants with LC; 50 variants with NI; 28 variants with ND; 8 variants with SI; 55 variants with AO; 1164 variants with RI; 3 variants with H and 2 variants with E, totally 31,038 variants (Fig. 2b). None of the variants is marked by only SSE. The tags LM (96%), LVF (88%), NI (86%) and ND (96%) are most effective, since almost all of variants marked by them are negative variants without confidence in benchmark. Above half of the variants with tags HDR (55%) and MV (69%) are also false positives out of the benchmark. LC (Low Coverage) may be an impediment to distinguish sequencing artifacts and considerably decline the confidence of a variant, thus, it also should be taken into key consideration, even though the proportion of negative variants marked by tag LC (21%) is not as high as other tags mentioned above. In order to recall these variants, a deeper depth sequencing data should be required. The other tags including RI (29%), RR (25%), SI (25%), AO (33%), MM (26%) and extremely DIR (13%) might not be effective for filtering variant, but they could reduce the confidence of the variant to some extent, which also requires comprehensive assessment.

#### Performance of variant filtering by *v-score*

**Fig. 3:**
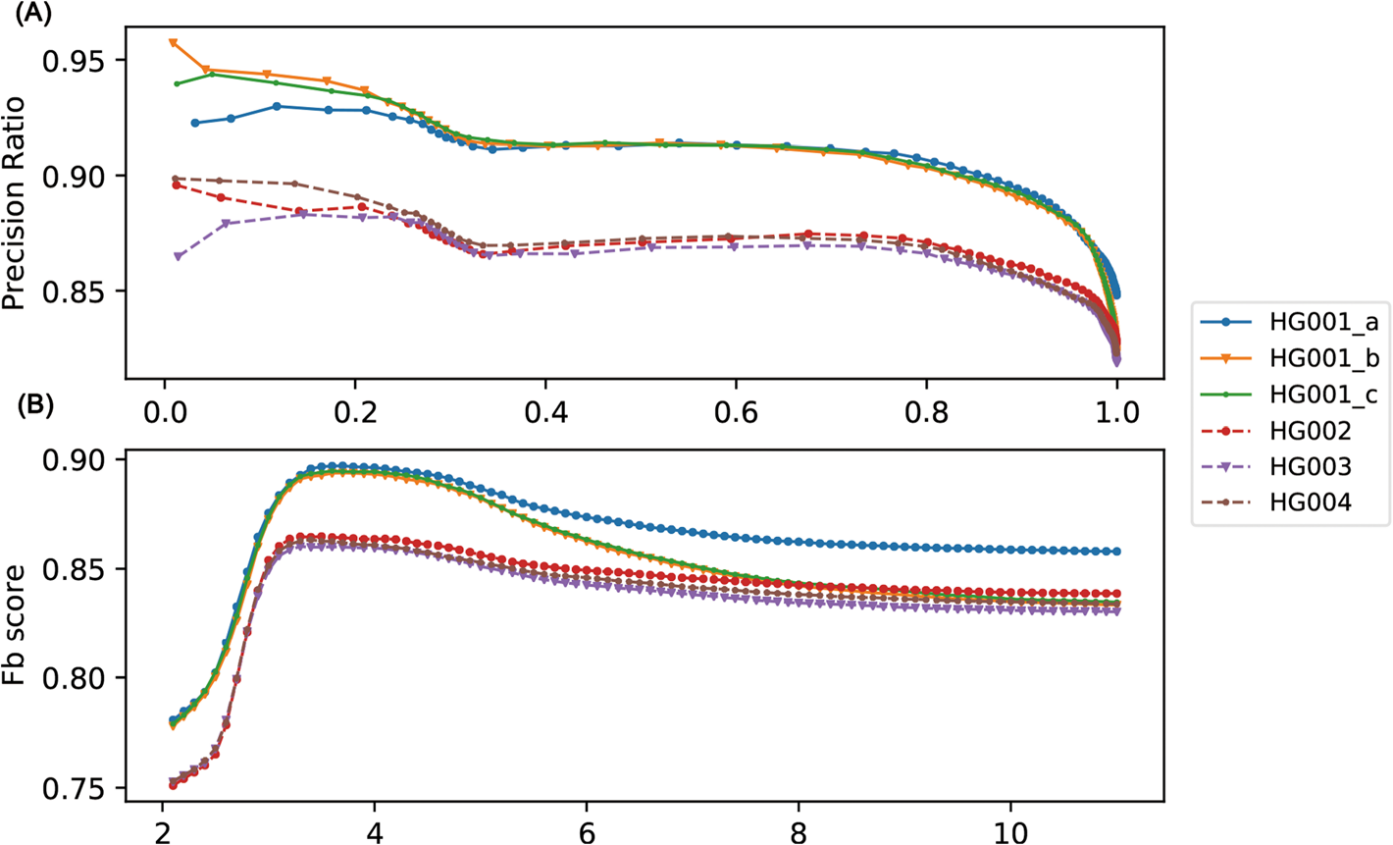
**a** R-P diagram plotting precision ratio versus recall ratio for six datasets. **b** Diagram plotting *F*_*β*_ Score versus *v-score*

We demonstrated the Recall-Precision diagram using the different *v-score* threshold with interval 0.1, as shown in Fig. 3a, and found that all samples exhibit similar trends. Making an overall consideration between precision ratio and recall ratio, *F*_*β*_ score (see Materials and Methods) was calculated for each dataset with *β* as 0.3. Figure 3b shows the relationship between *F*_*β*_ score and *v-score*. Interestingly, the *F*_*β*_ score of all datasets from the Illumina platform reaches the maximum when v-score is nearly 3.5 (Table 4). We also validated the performance of *v-score* for different *β* ranging from 0.1 to 0.5, and found that all *F*_*β*_ scores reach the maximum with the *v-score* from 3 to 4 (Additional file 2: Figure S2). It suggested that *v-score* is robust across different samples and choosing a threshold for *v-score* from 3 to 4 is reasonable. Notably, some complex variants might get high *v-score* due to the inaccuracy of alignment algorithm and sequencing error, which are still difficult to be detected.

**Table 4:** The performance of VariFAST for false positive variants filtering at the maximum *F*_*β*_ score for six standard sequencing datasets

ID	HG001_a	HG001_b	HG001_c	HG002	HG003	HG004
v-score	3.7	3.7	3.6	3.5	3.3	3.4
*F*_*β*_ score	89.7%	89.4%	89.5%	86.5%	86.0%	86.3%
Precision	90.4%	90.2%	90.4%	86.8%	86.6%	86.8%
Recall	82.5%	81.6%	80.1%	83.3%	80.0%	81.6%
Accuracy	77.8%	77.8%	76.8%	75.8%	73.6%	74.8%
MCC	0.3	0.38	0.37	0.22	0.23	0.23

#### Sanger sequencing validation for refined variants inconsistent with high confidence in benchmark

We selected 15 variants without high confidence in benchmark and with low *v-score*, including 7 SNVs, 4 insertions and 4 deletions to perform Sanger sequencing validation (Table 5). Almost all variants (14/15) were confirmed as true, which demonstrated that our *v-score* could efficiently recall a number of variants, although they are out of benchmark. There is only one variant failed in verification, as shown in the IGV visualization in Additional file 3: Figure S3. Three insert nucleotides were detected in locus 8,374,781 on chromosome 12. However, the result of Sanger sequencing discovered two SNVs (8,374,778: T > C; 8,374,786: G > T), which were extremely difficult to detected by both manual review and VariFAST.

**Table 5:** List of the information and result of Sanger sequencing validation

	Chromosome	Start	Ref	Alt	v-score	Tag	Validation
Without high confidence	Chr14	60,574,539	A	G	1.03		G
	Chr19	20,808,374	A	G	0.92		G
	Chr1	207,890,866	T	C	1.09		C
	Chr11	237,087	A	G	1.74		G
	Chr5	96,232,142	T	A	0.91		A
	Chr17	28,511,978	G	A	0.89		A
	Chr1	34,329,897	T	C	0.88		C
	Chr3	64,640,207	A	–	2.38		–
	Chr21	34,948,697	A	–	3.24		–
	Chr14	19,807,081	A	–	1.76		–
	Chr22	18,910,451	A	–	1.37		–
	Chr13	60,385,060	–	TTAC	1.21		TTAC
	Chr22	23,962,744	–	TTC	1.65		TTC
	Chr18	61,326,628	–	T	3.02		T
	Chr12	8,374,781	**–**	ACG	2.9		**–**
With high confidence	Chr10	25,273,311	–	A	11.7	LVF, D, MV, RR, RI	A
	Chr2	157,425,502	–	T	11.8	LVF, D, MV, RR, RI	T
	Chr8	101,725,036	–	AA	10.2	LVF, MV, RR, RI	AA
	Chr12	122,762,763	–	AA	10.0	LVF, D, MV, RR, RI	AA
	Chr6	82,930,437	–	A	7.0	LVF, D, MV, RR, RI	A
	Chr22	44,074,076	–	A	6.6	D, MV, RR, RI	A
	Chr17	7,012,073	G	T	6.5	HDR	T
	Chr15	81,624,768	C	T	5.3	LVF, LC	T
	Chr4	6,114,420	G	T	7.0	HDR	T
	Chr3	151,545,323	T	G	8.1	HDR, ND, D	G
	Chr1	89,652,094	G	A	7.0	LVF, HDR, H	A
	Chr11	55,595,114	G	T	6.7	HDR	T

We also chose other 12 variants with high *v-score* but within benchmark, which would be filtered by manual review obviously, to perform Sanger sequencing validation. All 12 variants even some with extremely low allele frequency were validated true. There are 5 variants marked by HDR were validated as Multiple Nucleotide Polymorphisms (MNPs). The visualization of MNPs were similar to HDR, whose variants were supported by reads that have other recurrent mismatches across the track (Additional file 4: Figure S4). Thus, *v-score* is difficult to distinguish these exceptional variants.

In conclusion, variants with low *v-score* can be regarded as true positive with considerable certainty. Oppositely, a few variants with higher *v-score,* which were usually also excluded by manual review, would be possibly misjudged. This is also the challenge for most of variant refinement methods.

#### Performance of variant filtering by machine learning model in VariFAST

**Fig. 4:**
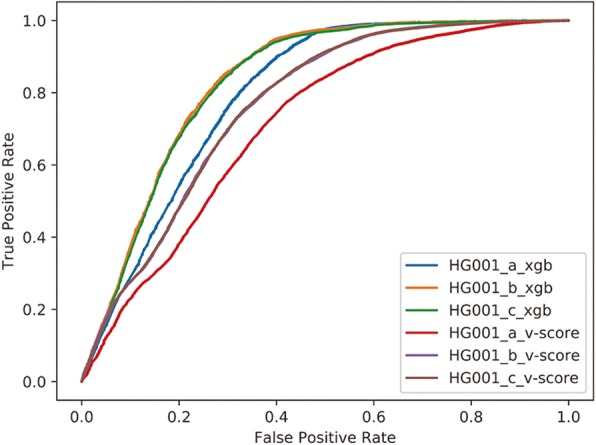
ROC curves plotting true-positive rate versus false-positive rate for three independent datasets predicted by XGBOOST model and *v-score* respectively

All variants (132534) called from three whole exome sequencing datasets (HG002, HG003, and HG004) were used to train the XGBOOST model. The hyperparameters were set as eta:0.1; gamma: 1; max_depth: 6 by ten fold cross validation for optimized grid search. Both *v-score* and XGBOOST were tested on three independent datasets sequenced by the Illumina platform (HG001_a, HG001_b, and HG001_c). The variants predicted as positive or negative consisted with annotation in benchmark are considered as true positive (TP) or true negative (TN). The variants predicted as positive but without high confidence in benchmark are considered as false positive (FP). Correspondingly, the variants predicted as negative but having high confidence of positive variants are considered as false negative (FN). The ROC curve [20] demonstrated that the XGBOOST model significantly performs better than *v-score* in all three datasets, as shown in Fig. 4. The AUC values are shown as follow: HG001_a: 0.790 vs 0.712; HG001_b: 0.832 vs 0.766; HG001_c: 0.828 vs 0.766. By using the var.roc function in R with bootstrap method, we got the variance of AUC are 1.66e-5, 7.52e-6, and 7.01e-6 respectively for datasets HG001_a, HG001_b, and HG001_c, which revealed the robustness of XGBOOST model.

In addition, we also used the datasets HG001_a, HG001_b, and HG001_c as training set and the datasets HG002, HG003, and HG004 as test set, the performance of XGBOOST is also similar.

### Validation on somatic variant filtering

#### Variant filtering using VariFAST for penile squamous cell carcinoma sequencing data

**Fig. 5:**
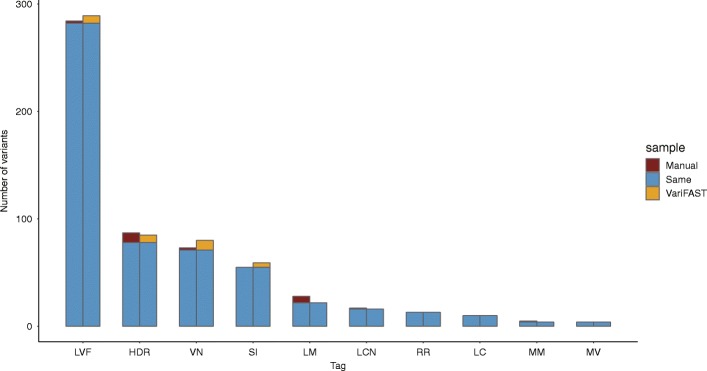
Comparison of the variants marked by VariFAST with manual review for penile squamous cell carcinoma sequencing data. Y coordinate represents number of variants marked with the corresponding tags listed on X coordinate. The legend ‘Same’ means the variant is marked by both manual review and VariFAST, ‘Manual’ and ‘VariFAST’ means the variant is marked by only manual review or VariFAST

Three paired samples from penile squamous cell carcinoma were sequenced to validate the consistency between our tool and manual review. Sequencing data were aligned to the reference genome (hg19) and 520 somatic variants located in functional regions were identified by GATK. We manually reviewed all variants using IGV according to the SOP proposed by [10] and marked tags for each variant, in which 158 variants were identified as high-quality somatic variants. Subsequently, we used VariFAST to calculate *v-score* and marked the tags for each variant. The results of VariFAST (Fig. 5) are highly consistent with the manual review, with 555 out of 603 tags are same. Specifically, 97% (289/291) LVF, 100% (10/10) LC, 94% (16/17) LCN, 93% (55/59) SI, 100% (13/13) RR, 86.6% (71/82) VN, 83% (78/94) HDR, 80% (4/5) MM, 78.6% (22/28) LM, 100% (4/4) MV marked by VariFAST are also identified by manual IGV review, while our pipeline saved massive time and labor. Importantly, the *v-score* of positive and negative variants groups are significantly different (*p*-value = 2.2e-16) under rank sum test (Additional file 5: Figure S5).

#### Variant filtering using VariFAST for pituitary adenomas sequencing data

**Fig. 6:**
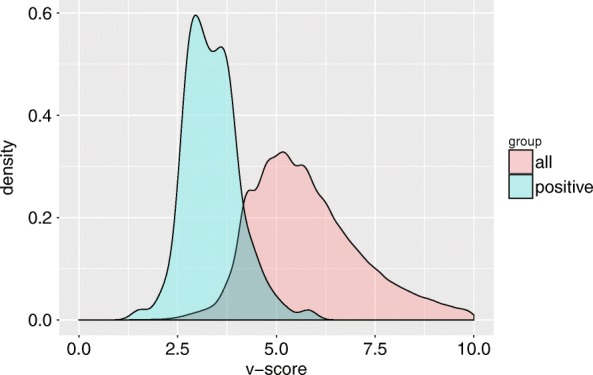
The *v-score* distribution of Sanger validated variants and total variants in pituitary adenomas samples. Legend ‘total’ (red) represents group of total variants, and ‘positive’ (green) represents group of Sanger validated variants

A large-scale whole exome sequencing data including 136 pituitary adenomas (PAs) cases published in our previous study was used for further evaluation of VariFAST for somatic variant refinement. Aggregately, 108,400 variants are called by GATK and 202 variants have been validated via Sanger sequencing. Figure 6 showed the distribution of *v-score* for both Sanger validated variants and total variants. The v-scores of almost all (174/202) validated variants are below 4, and the overall distribution of v-score for total variants showed significant difference with the validated subset (t-test, *p* < 10–16), which demonstrates the scoring by VariFAST is effective to distinguish positive and negative variations. Among the 202 validated true somatic variants, 169 variants are not marked with any tag contributing for false positive determination by VariFAST. The other 33 variants are marked by one unique tag, specifically 23 variants marked by LVF, 6 variants marked by DIR, 3 variants marked by LC, and 1 variant marked by LM. The parameters used for assigning tags for somatic variant filtering are: *thr*_*vaf*_ *=0.15*, *thr* =15, *thr*_*n*_ =5.

### Computation complexity of VariFAST

For each variant, VariFAST uses binary search to find reads covering the variant position across all tracks. The computation complexity is *MlogN*, where M is the number of tracks and N is the number of reads for one track. Then, VariFAST compared read with reference to find all other mismatches, the corresponding complexity is *LMlogN,* where L is the number of nucleotides for one read. Finally, for all variants, the complexity is shown as follows, where n is the number of variants.

17 $$ O\left(n,N,M,L\right)= nMLlogN $$

We provided an easy-to-use Python package for VariFAST implementation, which saves massive time and labor of manual review. The package uses ray [21], a distributed execution engine, to deal with task-parallel computations. It costs approximate 3 h for dataset HG001_a containing 30,000 germline variants with an average depth of 80, and 3 h for total 108,400 somatic variants from 136 PA cases with average depth of 90, using 64 cores on high performance computer. It can also be adapted to variant filtering of whole genome sequencing data with acceptable time. Our global pipeline integrating GATK Best Practices with VariFAST enables users to get high quality variants from raw sequencing data in more efficient way.

### Comparison of VariFAST with variant quality score recalibration (VQSR)

Variant quality score recalibration is the state-of-the-art variant filtering tool involved in GATK, it evaluates the probability that a SNP is a true genetic variant versus a sequencing or data processing artifact based on Gaussian mixture model. Several inevitable factors causing false positives including *near insertion rate, multiple variants rate*, have not been considered by VQSR. We compared the performance of *v-score* and XGBOOST model from VariFAST for true variant prediction with VQSR on public standard dataset HG001_a. VQSR was run with threshold 99% and the annotations including QD, SOR, FS, MQRankSum and ReadPosRankSum. Matthews correlation coefficient (MCC) and AUC were both calculated to evaluate the results of VQSR, XGBOOST model and *v-score*. XGBOOST model achieved the best AUC of 0.790, as compared to AUC of 0.712 for *v-score* and 0.756 for VQSR. XGBOOST model also obtained the best MCC of 0.522, compared to MCC of 0.295 for *v-score* and 0.488 for VQSR. In short, the machine learning model in VariFAST pipeline has better performance than VQSR, especially for INDEL variant filtering (Additional file 6: Figure S6). It because VQSR is expecting at least thousands of variant sites in order to achieve decent modeling with the Gaussian mixture model, while VariFAST is stable in the cases of variant filtering for small samples sequencing. The key characteristic of VariFAST is based on the comprehensive SOP summarized from experience of manual review, which is not affected by the sample size. The other important advantage of VariFAST is that the automated filtering by *v-score* can be used efficiently for both germline and somatic variants with better interpretability, while VQSR can not be used for somatic variants refinement. V-score is the sum of weighted metrics contributing for false positive variants, and the marked tags have been verified of biological significance accounting for the false positive.

## Discussion

Identification of high-quality variants is crucial to explore the in-depth view of genetic causes and find clinical treatment of disease. Although some variant discovery tools have been developed to call variants, such as GATK, there are still many false positive variants remain in final results. In order to reduce false positives, IGV-based manual review is required to filter variants, but it is very time-consuming and usually has personal preference. Here, we developed an automated approach VariFAST, and provided an easy-to-use tool, to help researchers solve the problem. We also provided a pipeline integrating GATK Best Practices with VariFAST, which can be easily used for high quality variants detection from raw data. VariFAST runs dramatically faster than manual review, nearly 3 h for 30,000 germline variants with average coverage of 80 using 64 cores computation, which can free researchers from heavy manual work. VariFAST tool possesses high flexibility, which enables researchers to set different parameters for application in particular studies.

### Scoring by quantitative metrics and tags determine the good performance of VariFAST

We proposed 18 quantitative metrics based on the SOP for IGV manual review [10], and assigned different weights on metrics from level 1 to 3 according to their importance. Then VariFAST calculates *v-score* as the sum of weighted metrics, which is robustly from 3 to 4 under the maximum *F*_*β*_ score indicating both good precision and recall (Additional file 2: Figure S2). Next, VariFAST marks tags for each variant according to the standard in Table 1, we identified the top effective tags for determining false positive variants, including LM, LVF, NI and ND, LC (Low coverage) should also be taken into key consideration. Correspondingly, the variants having no tag are possibly true positives. In addition, almost all of the positive variants determined by low *v-score* but without high confidence in benchmark have been validated as true positive by Sanger sequencing validation (Table 5), which indicated that the automated scoring based on quantitative metrics and tags can recall a number of variants that were easily filtered out.

### VariFAST is effective for both germline and somatic variants filtering

VariFAST has been validated useful for both germline and somatic variants refinement. We performed VariFAST on 279,573 germline variants called from six sequencing datasets of standard samples (NA12878) and discovered that all datasets from Illumina platform have similar optimal *v-score* thresholds (Fig. 3b) determined by *F*_*β*_, which means v-score is robust in application for different sequencing datasets. The Sanger sequencing validation of HG001 demonstrated high accuracy of VariFAST for positive recall. In addition, two independent cancer datasets were used to validate the performance of VariFAST to refine somatic variants. For penile squamous cell carcinoma sequencing data, we found the result of VariFAST are highly consistent with the manual review (Fig. 5). For sequencing data of 136 pituitary adenomas samples, we showed the *v-score* distribution of Sanger validated variants is significantly lower than that of the total variants, which means the scoring by VariFAST is effective to distinguish positive and negative variations.

### XGBOOST model in VariFAST outperforms state-of-the-art VQSR

VariFAST also provides a XGBOOST model trained using the datasets of Ashkenazim Trio from Illumina platforms for germline variant filtering. XGBOOST model generated an average AUC of 0.82 using three HG001 datasets as test set, which performed better than *v-score* (Fig. 4). However, XGBOOST model could not be used widely across different types of sequencing platforms due to the bias of training data. Furthermore, compared with *v-score*, XGBOOST model may have worse interpretability, because no specific tags will be marked to explain why the variant is positive or negative. Therefore, we suggest that the combination of XGBOOST model and *v-score* should be all taken into consideration for germline variants filtering.

By comparison with state-of-the-art variant filtering method VQSR in GATK, XGBOOST model reveals better MCC and AUC, especially more superior for INDEL variants filtering, because it is based on the comprehensive SOP summarized from experience of manual review. Moreover it has no prerequisite of large scale samples and variant sites opposed to VQSR. Although both VQSR and XGBOOST model have limitations in somatic filtering, the *v-score* of VariFAST has potential to be the dominant tool for discovering true positive somatic mutation for disease study.

### Further work

There are still some limitations for VariFAST. Firstly and most importantly, complex variant such as Multiple Nucleotide Polymorphisms, which is similar to HDR, is difficult to detect, so more complex metric may be required to be proposed. Secondly, the weights of metrics are difficult to determine for different tasks due to the absence of gold standard datasets, assigning reasonable weights on each metric by auto-learning will be considered in our further work.

## Conclusions

We developed a novel approach VariFAST for high quality variants detection from raw data, which includes the *v-score* part by sum of weighted metrics causing false positive variations and the predictive model part trained by XGBOOST algorithm for germline variants refinement. VariFAST is an automated and efficient approach for both germline and somatic variant filtering, which is promising to substitute for the laborious IGV manual review.

## Supplementary information

**Additional file 1: Figure S1.** Flowchart of the whole pipeline integrating GATK Best Practices with VariFAST. The part of the dotted frame is our tool VariFAST.

**Additional file 2: Figure S2.** Diagram plotting *F*_*β*_ Score versus *v-score* with different β. The β of upper diagram is 0.1 and the below one is 0.5.

**Additional file 3: Figure S3** The visualization of variant at location 8,374,781 on chromosome 12 in dataset HG001_a.

**Additional file 4: Figure S4.** The visualization of variant at location 55,595,114 on chromosome 11 in dataset HG001_a.

**Additional file 5: Figure S5.** The violin plot of positive and negative groups. The F means negative groups and S means positive groups.

**Additional file 6: Figure S6.** The ROC curve for *v-score*, XGBOOST model and VQSR in INDEL variant filtering on dataset HG001_a.

## Data Availability

Data set of HG001_a is available on the ftp site of 1000 genomes (ftp://ftp.1000genomes.ebi.ac.uk/vol1/ftp/). Data sets of HG001_b and HG001_c are sequenced by our lab. Data sets of Ashkenazim Trio including HG002, HG003 and HG004 are available on the ftp site of GIAB (ftp://ftp-trace.ncbi.nlm.nih.gov/giab/ftp/data/). The VariFAST source code and the pipeline integrating with GATK Best Practices are available at https://github.com/bioxsjtu/VariFAST.

## Abbreviations

AUC: Area under curve
GATK: Genome Analysis ToolKit
IGV: Integrative Genomics Viewer
MCC: Mathew correlation coefficient
VQSR: Variant Quality Score Recalibration
